# Supplementary material for: FERN – a Java framework for stochastic simulation and evaluation of reaction networks
Source: BMC Bioinformatics. 2008 Aug 29;9:356. doi: 10.1186/1471-2105-9-356 (PMC2553347; doi:10.1186/1471-2105-9-356)
Supplement: Additional file 1 — FERN distribution, Version 1.3. This archive contains the FERN source code and binaries as well as documentation and example models in FernML and SBML. [file 1471-2105-9-356-S1.zip › fern/doc/javadoc/fern/network/sbml/MathTree.html]

MathTree


---


|  |  |  |
| --- | --- | --- |
| **Overview** \| **Package** \| **Class** \| **Use** \| **Tree** \| **Deprecated** \| **Index** \| **Help** | |  |
| PREV CLASS   **NEXT CLASS** | **FRAMES**    **NO FRAMES**     **All Classes** |  |
| SUMMARY: NESTED \| FIELD \| CONSTR \| METHOD | DETAIL: FIELD \| CONSTR \| METHOD |  |


---


## fern.network.sbml Class MathTree

```
java.lang.Object
  fern.network.sbml.MathTree
```

---

``` public class MathTree extends Object ```

Representation of am evaluation tree. Within a sbml file, MathML branches may occur at
different positions. These are represented as MathTrees in FERN.

**Author:**
:   Florian Erhard

---

| **Nested Class Summary** | |
| --- | --- |
| `static class` | `MathTree.ConstLeaf` |
| `static class` | `MathTree.GlobalLeaf` |
| `static class` | `MathTree.InnerNode` |
| `static class` | `MathTree.Node` |
| `static class` | `MathTree.VarLeaf` |


**Constructor Summary**

`MathTree(Network net,
org.sbml.libsbml.ASTNode ast,
Map<String,Double> globals,
Map<String,Double> locals,
Map<String,Integer> bindings)`
  
          Creates a MathTree from an libsbml `ASTNode`.


| **Method Summary** | |
| --- | --- |
| `double` | `calculate(AmountManager amount, Simulator sim)`             Evaluate the MathTree. |
| `MathTree.Node` | `getRoot()`             Gets the root of this MathTree. |
| `List<Integer>` | `getSpecies()`             Gets the species present in this tree. |
| `static String` | `inferStringFromSBMLConstant(String prefix, Object c)`             Uses reflection to get a string representation of a libsml constant. |

**Methods inherited from class java.lang.Object**

`clone, equals, finalize, getClass, hashCode, notify, notifyAll, toString, wait, wait, wait`

**Constructor Detail**

### MathTree

```
public MathTree(Network net,
                org.sbml.libsbml.ASTNode ast,
                Map<String,Double> globals,
                Map<String,Double> locals,
                Map<String,Integer> bindings)
```

:   Creates a MathTree from an libsbml `ASTNode`.

    **Parameters:**: `net` - sbml network: `ast` - ASTNode: `globals` - pointer to the global variable mapping: `locals` - pointer to the local variable mapping of this entity: `bindings` - mapping of the variable names to their indices


**Method Detail**

### getSpecies

```
public List<Integer> getSpecies()
```

:   Gets the species present in this tree.

    :   **Returns:**: indices of the species.

---


### getRoot

```
public MathTree.Node getRoot()
```

:   Gets the root of this MathTree. Feature versions may implement the Visitor pattern,
    but for now traversing by using instanceof will do.

    :   **Returns:**: the root of this MathTree

---


### calculate

```
public double calculate(AmountManager amount,
                        Simulator sim)
```

:   Evaluate the MathTree.

    :   **Parameters:**: `amount` - AmountManager: `sim` - Simulator **Returns:**: value of the expression

---


### inferStringFromSBMLConstant

```
public static String inferStringFromSBMLConstant(String prefix,
                                                 Object c)
```

:   Uses reflection to get a string representation of a libsml constant.

    :   **Parameters:**: `prefix` - a prefix for the constant name e.g. AST: `c` - the libsml constant **Returns:**: the name of the constant in libsbmlConstants


---


|  |  |  |
| --- | --- | --- |
| **Overview** \| **Package** \| **Class** \| **Use** \| **Tree** \| **Deprecated** \| **Index** \| **Help** | |  |
| PREV CLASS   **NEXT CLASS** | **FRAMES**    **NO FRAMES**     **All Classes** |  |
| SUMMARY: NESTED \| FIELD \| CONSTR \| METHOD | DETAIL: FIELD \| CONSTR \| METHOD |  |


---
